# Thermography-Based Deterioration Detection in Concrete Bridge Girders Strengthened with Carbon Fiber-Reinforced Polymer

**DOI:** 10.3390/s20113263

**Published:** 2020-06-08

**Authors:** Van Ha Mac, Jungwon Huh, Nhu Son Doan, Geunock Shin, Bang Yeon Lee

**Affiliations:** Department of Architecture and Civil Engineering, Chonnam National University, Gwangju 61186, Korea; 188456@jnu.ac.kr (V.H.M.); 188444@jnu.ac.kr (N.S.D.); gos3452@jnu.ac.kr (G.S.); bylee@jnu.ac.kr (B.Y.L.)

**Keywords:** concrete strengthened with CFRP (CSC), concrete without CFRP (CWC), concrete girder, bridge, delamination, step heating thermography, infrared thermography, non-destructive testing

## Abstract

In bridge structures worldwide, carbon fiber-reinforced polymer (CFRP) sheets are applied to strengthen weak components, especially concrete girders that are at a high risk of rapid degradation during the bridge’s operation owing to impacts from the superstructure’s weight and traffic loads. Regarding the thermography-based method (TM), although deteriorations in the concrete core are some of the main defects in concrete structures strengthened with CFRP, these do not receive as much attention as damage in the CFRP. Therefore, the interpretation of the structural health in terms of these defects using TM is still unclear. The problem presented in this work addresses the quantification of delamination inside the concrete part of a specimen with a CFRP sheet installed on the surface (assumed to be the girder surface strengthened with CFRP) via step heating thermography. Additionally, the empirical thermal diffusivity of concrete girders strengthened with a CFRP sheet (CSC girder), has not been provided previously, is proposed in the present study to predict delamination depths used for field investigations. Moreover, the effect of the CFRP sheet installed on the structure’s surface on the absolute contrast of delamination is clarified. Finally, advanced post-processing algorithms, i.e., thermal signal reconstruction and pulsed phase thermography, are applied to images obtained with step heating thermography to enhance the visibility of delamination in CSC girders.

## 1. Introduction

The aging of structural components is one of the most critical factors leading to the deterioration of concrete bridges [1]. In Korea, the proportion of bridges over 30 years old was 30.9% in 2015 and this value will increase to 70.2% by 2025 [2]. In the United States, 72% and 38% of bridges listed in the National Bridge Inventory were over 25 and 50 years old in 2015, respectively [3]. In Japan, 18% of bridges were over 50 years old by 2013 and this number will increase to 43% in 2023 and 67% in 2033 [3]. Therefore, extending the lifetimes of bridges is a crucial task.

Defects in the concrete structures in the forms of cracks, delamination, and spalls are unavoidable and mainly caused by rebar corrosion [4,5]. The worldwide cost for the maintenance and repair of concrete structures affected by the corrosion amounts to millions of dollars each year, as reported by Zhang et al., in 2019 [6]. In bridges, the girder supports the entire weights of the superstructure and traffic loads. Thus, the abovementioned defects can occur frequently in concrete girders, especially in bridges located near the sea or in areas at a high risk of chloride, moisture, and oxygen penetration [7].

**Figure 1:**
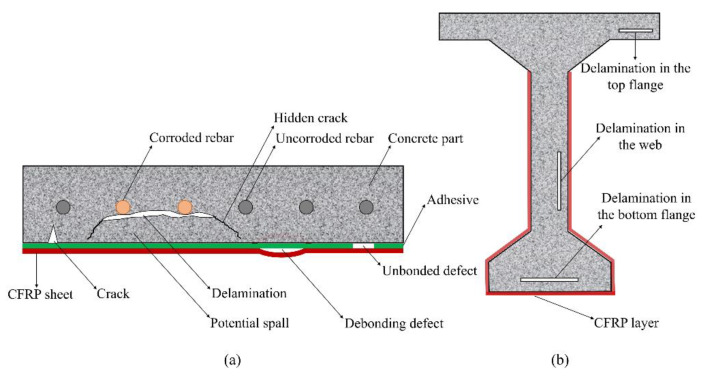
Deteriorations forming in concrete structures strengthened with one carbon fiber-reinforced polymer (CFRP) layer: (**a**) common types of defects; (**b**) delamination in T-shaped girders.

Many methods have been applied in recent times to strengthen the weak components of bridges with the purpose of meeting the traffic demand [8,9]. Of these methods, carbon fiber-reinforced polymer (CFRP) has become an efficient and economical technique for strengthening concrete girders [9,10]. However, deteriorations will inevitably develop at the core of the concrete part of girders strengthened with CFRP during the bridge’s operation. The normal stresses developed in a bonded CFRP layer can cause delamination, as stated in ACI 440.2R-02 [11]. In addition, this is also because the development of the rebar corrosion in girders caused by chloride, moisture, and oxygen penetration cannot be entirely avoided. Furthermore, although potential defects in the girder must be repaired before CFRP sheets are installed, damage can also develop owing to a weak connection between the old and new parts in the mended regions. Moreover, defects might form in the concrete part without CFRP and then spread to the area strengthened with CFRP. Undetected defects can exist in the girder prior to the preparation process for applying the CFRP. In concrete strengthened with one CFRP layer, defects can form both in the concrete and CFRP parts as shown in Figure 1a [12].

Among the defects inside the concrete part, delamination that mainly develop within the concrete cover above the rebars are usually invisible under visual inspection even after the CFRP sheets are removed. Such delamination can lead to potential spalls as described in Figure 1a [12,13]. Therefore, this kind of defect needs to be detected as quickly and accurately as possible before it causes any serious damage to the concrete girder. Figure 1b illustrates an example of a concrete T-shaped girder (a common type in concrete bridges) with delamination in the core of the concrete areas strengthened with CFRP and those without CFRP.

Non-destructive testing (NDT) methods have been utilized to accurately indicate internal defects of the concrete structures [14]. Although a large number of developments have been created related to NDT investigation up to the present, there is still no international standard NDT methods applied to concrete bridges [15]. In terms of Standard for infrared thermography (IRT) methods to detect defects in concrete bridges, one named ASTM D4788-03 “Standard Test Method for Detecting Delamination in Bridge Decks Using Infrared Thermography” was published until now [16]. However, this standard gives requirements for only the passive approach (the sunlight is used as the heat source) to detect delamination of the concrete bridge deck. There seems to be no standard for active IRT (artificial heat sources are employed) so far. Thus, the study of the applicability of this method to detect defects not focused in previous works in components of bridge structures (e.g., concrete girders strengthened with CFRP) is necessary.

Pulsed thermography (PT) and step heating thermography (SHT) or so-called long pulse thermography are two popular methods in active IRT [17,18]. From a mathematical point of view, the thermal signal contains exactly the same information in PT and SHT. The difference between the two methods above is that a short thermal stimulation from a few milliseconds to a few seconds is provided in PT whereas the heating time is from a few minutes to hours in the SHT method [18]. Therefore, PT is usually used for the material with a high thermal conductivity like metal and CFRP or shallow defects while SHT is interested in the case of a small thermal conductivity, e.g., concrete or deep defects. The halogen lamp is one of the most effective external heat sources alongside others such as the infrared heater and flash lamp used in aforementioned methods. Generally, the structure surface is heated by the external heat source and the surface temperature of structure is recorded during heating and/or cooling time (after the heat source is turned off) in both PT and SHT methods. However, in PT and SHT methods, the cooling time is more important in comparison with heating time to extract the defect information. Up to the present, the SHT has been proven as an effective NDT method to identify not only delamination but also voids in concrete structures without CFRP (CWC structures) [19,20].

For concrete structures strengthened with CFRP (CSC structures) focused in this study, PT is an efficient method utilized to detect accurately defects in the CFRP part or surface defect of the concrete part. Tashan et al. in 2014 [12] conducted tests on four CSC specimens using PT method with the heat source as halogen lamps. Three types of deteriorations in the CSC structure including unbonded defects, debonding defects, and delamination within the CFRP part were investigated. The authors concluded that a lengthened pulse could produce a greater contrast between the area above a defect and its surroundings. Furthermore, they recommended that the input heat flux and pulse length should be higher than 500 W/m^2^ and 1 s, respectively. In addition, the maximum thermal signal decreases under an increase in defect depth. Moreover, in 2014, Tashan et al. [21] focused on the defect detection in the concrete substrate part of a CSC beam, i.e., cracks employing PT method with the heat excitation of halogen lamps. This work demonstrated that the PT method can accurately identify the location and size of major cracks (of up to 0.8 mm in size). The best crack detection was obtained with a 5-s pulse length. Additionally, the SHT was applied to identify defects in concrete structures strengthened with two CFRP layers by using two red-ray heaters as the heating source in the work conducted by Gu et al. in 2020 [22]. Four specimens were made in which the CFRP (two layers with the density of 200 g/m^2^) and cover mortar (one layer with 10 mm of thickness) were designed above the concrete specimen. Different square artificial defects with the size ranging from 5 to 20 mm were implanted on the concrete surface and at the interface between the CFRP and cover mortar. The locations and shape of defects are accurately detected and determined, respectively, by using the proposed experimental setup. In addition, the influence of defect size and depth, the water inside defect and cover mortar were indicated in this study. Otherwise, an unusual heat source, i.e., a hot pack with a nearly constant surface temperature of 40 °C, was utilized for the IRT test in the study [23] in which artificial defects at the interface of CFRP and concrete beam surface were the targets. The accuracy of the expected size of defects in this work was about 88%. Focusing only on the CFRP structure, in 2016, Liu et al. [24] carried out a test on a CFRP specimen that was composed of eight CFRP plies with the same thickness of 0.42 mm. The results indicated that shallow defects (depth ≤ 2.11 mm) at the interfaces of CFRP plies can be detected using the PT method with the heat excitation of two halogen lamps. Furthermore, active IRTs were also applied in the defect detection in CSC structures in many recent studies such as Shardakov et al. in 2016, Shi et al. in 2019, Wu et al. in 2019, and Lai et al. in 2020 [25,26,27,28].

Although the active IRT methods, i.e., PT and SHT, have been utilized worldwide to identify defects in CSC structures not exposed directly to sunlight, previous investigations usually focused on defects in the CFRP part or on the concrete surface, while defects at the core of the concrete part, e.g., delamination, received much less attention. Additionally, CFRP is a technique applied commonly to strengthen the concrete girders that are important but vulnerable parts of bridges. Therefore, the capability of NDT methods such as SHT in the quantitative detection of damages inside the concrete part of CSC girders is an unclear issue that needs to be studied further. Because, this study focuses on delamination located up to 8 cm from the concrete surface (material with a small thermal conductivity) of CSC structures, SHT method is employed as the detection method. Meanwhile, the thermal diffusivity of CSC girders used in the prediction of delamination depths in the SHT method has not been proposed in previous studies; therefore, this factor is experimentally determined in this work. Furthermore, an examination of effects of the CFRP sheet installed on the concrete surface on contrast values of delamination is conducted. Moreover, every pixel of the thermal image sequence obtained with SHT method is post-processed using two advanced algorithms involving thermal signal reconstruction and pulsed phase thermography to enhance the accuracy of the detection of delamination in CSC girders. A clarification of the aforementioned issues can contribute to improving the effectiveness of field investigations of concrete bridges.

## 2. Methods

The STH method is considered as time-resolved infrared radiometry [18]. The terms of time-resolved indicates that the temperature is monitored during both heating and cooling time. In addition, methods employing pulsed heating could also be taken into consideration as time-resolved [18]. Thus, the theory of pulsed heating response is presented in Section 2.1. Under the pulsed heating, the thermographic data provides some information about the presence of defects because of the surface temperature difference between areas above delamination and its surrounding.

Nowadays, many processing algorithms have been applied to improve the quality of the results from the IRT method. These algorithms include simple methods such as contrast, pseudo-color images, histogram equalization and image filters, and other advanced methods, like pulsed phase thermography, thermal signal reconstruction, principal component thermography, slope and R^2^ and dynamic thermal tomography [29,30]. In the present study, a simple algorithm, i.e., absolute contrast and two advanced algorithms, i.e., pulsed phase thermography and thermal signal reconstruction are applied to process the data from the SHT method. In addition, the definition of signal-to-noise criterion is also given that is used in conjunction with the absolute contrast value to assess the defect detection in this work. The definition of these algorithms is presented in Section 2.2, Section 2.3, and Section 2.4.

### 2.1. Pulsed Heating Response

The surface temperature of a semi-infinite homogeneous and isotropic solid under pulse heating with the magnitude of *I*_0_ (J/m^2^) can be determined using Equation (1) [18,31,32,33]:

(1) Tnde=T0+I0e×(π×t)12

where *T_nde_* is the surface temperature at time instant *t*, *T_0_* is the initial surface temperature, *e* (Ws^1/2^m^−2^K^−1^) is the thermal effusivity, and *α* (m^2^/s) is the thermal diffusivity.

If a defect such as a delamination develops inside the structure at the depth of *z_d_*, the structure’s surface temperature above this defect can be determined by Equation (2) [31]:

(2) Tde=T0+I0e×(π×t)12×[1+2∑n=1∞Rn×exp(−(n×zd)2α×t)]

where *R* is the effective thermal reflection coefficient of the interface between the structure material and air and is close to 1 [31]. *n* is the mathematical form of the pulse reflections from the interface between the air inside the defect and structural material. The surface temperature above a defect is higher than that of its surroundings, as shown in Equation (3). Thus, the defect can be observed on thermal images captured by IR detectors.

(3) ΔT(t)=Tde−Tnde=I0e(π×t)12×2∑n=1∞Rn×exp(−(n×zd)2α×t)

It should be noted that the theory mentioned above is based on ideal assumptions. The heat transfer and surface temperature response, in reality, are very complicated, especially in the case of CWC (highly heterogeneous material) and CSC (a combination of several materials). In this research, the surface temperature response of CWC and CSC structures captured by an IR detector is not theoretically considered in the form of mathematic but is experimentally studied in the laboratory.

### 2.2. Thermal Contrast and Signal-to-Noise Computation

In active IRT, four traditional contrast definitions, including absolute, running, normalized, and standard contrasts, are commonly employed to quantitatively evaluate the defect detection on thermal images [12,13,14,18,34,35,36,37,38,39,40]. In addition, another contrast method called differential absolute contrast (DAC) was proposed and applied successfully to investigate the structural health [41,42]. In the DAC method, the manual selection of a non-defected area is not necessary.

Among the methods described above, the running and normalized contrasts are applied to reduce the disadvantages of the absolute and standard contrasts in which the effects of a non-uniform surface and emissivity can be reduced [18,36,43,44,45]. The drawback of the normalized contrast is that the time instant of maximum excess temperature must be determined first [18]. Furthermore, the dependence of the absorbed energy is not significant in the running contrast [18]. However, determining the effect of the heating time in relation to the energy absorbed by the specimen is also a main task in this study. Additionally, proper selection of the time at which the first defect becomes visible is the main challenge in the DAC method. Thus, for simplicity, the absolute contrast method is employed for analyzing the data and to consider the defect detection. In the absolute contrast method, the thermal contrast and observation time, which are two major indicators used to discover defects and predict their depths, can be obtained. The absolute contrast (*C^ab^*(*t*) or Δ*T*(*t*)) determined using Equation (4) was also efficiently applied in the IRT method to analyze the thermal images in previous works [21,46,47,48]:

(4) $$C^{ab}\left(t\right)=\Delta T\left(t\right)=T_{de}\left(t\right)-T_{nde}\left(t\right)$$

where *T_de_*(*t*) and *T_nde_*(*t*) are the surface temperatures above the delaminated and non-delaminated areas at time instant *t*, respectively. The selection of areas used to calculate *T_de_*(*t*) and *T_nde_*(*t*) is discussed in Section 4.1.

Previously, signal-to-noise ratio criterion shown in Equations (5) and (6) is frequently used to indicate the contrast goodness of delaminations on the thermal images [14,49]. Therefore, the SNR is chosen to evaluate the defect detection in conjunction with the absolute contrast value as well as appraise the efficiency of pulsed phase thermography and thermal signal reconstruction in this study. It should be noted that the greater is the SNR, the more clearly is the defect identified.

(5) $$SNR=20\times {log}_{10}\left(\frac{\left|S-N\right|}{\sigma_{noise}}\right),$$

(6) $$\sigma_{noise}=\sqrt{\frac{1}{n-1}\times {\sum_{i=1}^{n}\left(x_{i}-x_{average}\right)}^{2}}$$

where *S* and *N* are the average surface temperatures above a defect and its surroundings, and $\sigma_{noise}$ is the standard deviation of the surface temperature around the delamination. The selection of areas used to calculate *S*, *N*, and $\sigma_{noise}$ is shown in Section 4.1

### 2.3. Post-Processing Technique: Thermal Signal Reconstruction

The temperature change on the surface of a structure after heating at a time instant *t* can be shown as per Equation (7), which is based on Equation (1) [32,50]. A double logarithmic scale of Equation (7) is considered and Equation (8) is obtained.

(7) ΔT(t)=Tnde−T0=I0e(π×t)12

(8) $$ln\Delta T\left(t\right)=ln\left(\frac{I_{0}}{e\sqrt{\pi }}\right)-\frac{1}{2}\times lnt$$

As indicated in Equation (8), the logarithmic line of the temperature change has a slope of 0.5. However, the experimental curve usually varies depending on many factors such as the technical specifications of the IR camera, reflection of artifacts, and convection phenomena. Therefore, thermal signal reconstruction (TSR) is frequently employed to overcome these problems [30,32,50]. In TSR, the correlation between *ln*Δ*T*(*t*) and *lnt* is approximated by a polynomial line with a degree of n, as given in Equation (9):

(9) $$ln\left(\Delta T\left(t\right)\right)=\sum_{i=0}^{n}\left(a_{i}\times {\left(lnt\right)}^{i}\right)$$

where coefficients *a_i_* are determined using the least squares regression. In previous studies, the degree *n* was usually selected as 4 or 5, which is an optimal choice to obtain the best noise reduction and data fitting [30,32]. Hence, a sequence of thermal images is replaced with *n* + 1 images of coefficients. Typically, the first and second derivatives of Equation (9) are used directly to reduce the noise of the raw data. In the present study, the TSR method is applied to the thermographic sequence after lamp heating (during the cooling time) with the degree *n* equal to 5.

### 2.4. Post-Processing Technique: Pulsed Phase Thermography

Pulsed phase thermography (PPT), a process in which the temperature is transformed from a time domain to a frequency domain, is widely utilized to qualify defects in structures [51]. PPT combines the advantages of PT’s rapid inspection and lock-in thermography’s extraction of phase delay information [52]. The fast Fourier transform can be used as the transform method in PPT and applied to each pixel using Equation (10) [30,52]:

(10) Fn=Δt×∑k=0N−1T×(k×Δt)×exp(−j2πnkN)=Ren+jImn

where Δ*t* is the sampling interval, *N* is the total number of thermal images, and *n* is the frequency increment. The initial results of the transformation are *Re_n_* and *Im_n_*, corresponding to the real and imaginary parts. Finally, the amplitude (*A_n_*) and phase (*φ_n_*) images are obtained using Equations (11) and (12) for every pixel that can reduce the temporal noise of the raw data [30,52].

(11) $$A_{n}=\sqrt{{\left({Re}_{n}\right)}^{2}+{\left({Im}_{n}\right)}^{2}}$$

(12) $$\varphi_{n}=arctan\left(\frac{{Re}_{n}}{{Im}_{n}}\right)$$

## 3. Experimental Procedure

### 3.1. Specimen Implementation

**Figure 2:**
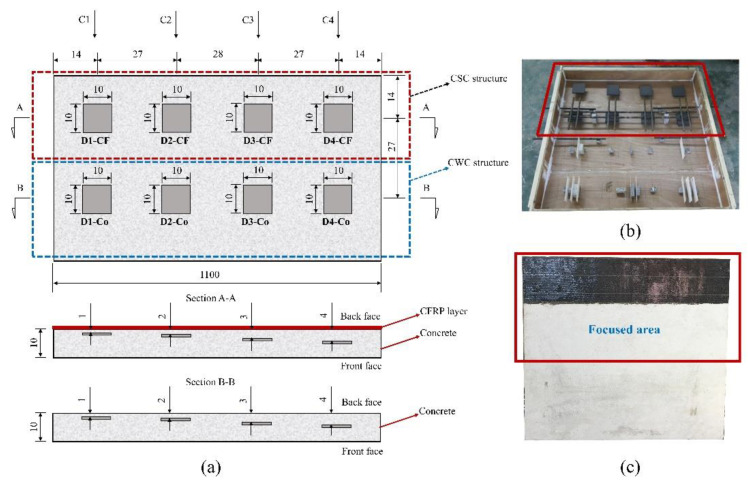
Specimen of concrete strengthened with CFRP (CSC) and concrete without CFRP (CWC): (**a**) arrangement of embedded delaminations; (**b**) formwork of specimen; (**c**) CFRP sheet installed on the back-face of specimen.

One concrete specimen with a design compression strength of 30 MPa was cast. At the core of the specimen, eight squared artificial delaminations with the same size of 10 cm × 10 cm × 1 cm were implemented, as shown in Figure 2a. The artificial delaminations were located at depths of 1, 2, 3, and 4 cm from the back-face and 5, 6, 7, and 8 cm from the front-face. These delaminations were made by polystyrene with the thermal conductivity about k = 0.027 W/m°C that is similar compared to the air k = 0.024 W/m°C [48,53]. In addition, artificial defects were attached on the surface of stone pieces (made from the concrete with the same mixture ratio compared to the specimen), then stone pieces were glued on the bottom wooden plate of formwork. Thus, delaminations with exact depths as designed are created. Therefore, the specimen and artificial delaminations are expected to have similar response under heat transfer in comparison with real concrete bridge deck structures. Figure 2b,c depict the formwork in the specimen fabrication process and the CFRP installation, respectively.

Actually, the selection of the CFRP system to strengthen concrete structures depends on many factors such as CFRP properties, the structure condition and purpose of strengthening [11]. In this study, the system with a single CFRP layer was selected with the assumption that it was installed perfectly on the structure surface. To prepare the specimen, a one-direction CFRP sheet with a thickness of 0.167 mm was installed at the upper part of the specimen, as shown by the red dashed rectangle in Figure 2a, which represents the surface of the CSC girder. The lower part within the blue dashed rectangle in Figure 2a stands for the surface of the CWC girder. In each column (C1, C2, C3, and C4 in Figure 2a), there were two delaminations (one in the CSC part and one in the CWC part) with the same parameters, i.e., size, depth, and thickness, which allows for consideration of the CFRP effect on the detection of delaminations at the core of concrete. The characteristics of artificial delaminations are listed in Table 1.

The CFRP sheet was installed as per the six following steps. First, the concrete specimen surface was carefully cleaned using a handheld grinding machine. Then, the primer epoxy coat was applied on the concrete surface. Thereafter, the main epoxy coat was enforced on the primer layer. Following this, the pre-cut CFRP sheet was installed by hand, and a regular paint roller was used to press the fabric on the concrete surface. Finally, an additional epoxy coat was applied to the installed CFRP sheet.

### 3.2. Experimental Work

In the present study, a series of experiments was conducted using the SHT method. First, the CFRP sheet was applied on the back-face and tests were conducted from this face. Thereafter, the CFRP on the back-face was removed, the other CFRP sheet was installed on the opposite face, and remaining tests were conducted from the front-face.

**Figure 3:**
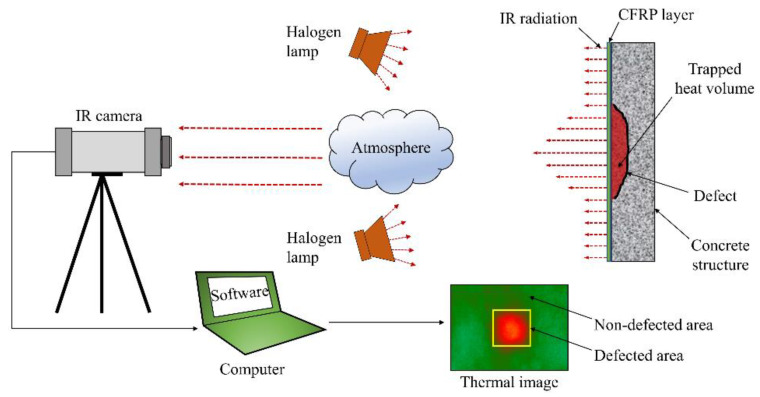
Experimental scheme.

The specimen surface was heated using an artificial heat source with a total energy of 2000 W, provided by a set of four halogen lamps (500 W per lamp). It should be noted that the halogen lamp is a commonly used heat source in the SHT method. A steel frame was employed to fix the halogen lamps 1.2 m from the specimen surface. The arrangement of the equipment is shown in Figure 3.

In fact, because of the physical restriction of lamps and low thermal conductivity of concrete, the structure is usually required to be heated using a long heating time since the surface structure can achieve the needed energy input to detect deep delaminations [54,55]. However, if the heating time is too long, i.e., around one hour, the thermal contrast above a defect might degenerate [55]. The optimal heating time can be evaluated under a certain depth and a known thermal diffusivity, but it is hard to be in the know before the experiment is conducted [55]. In addition, the deeper the delamination the longer the heating time needs to be provided. Therefore, in this paper, the different heating regimes were provided for the back- and front-face tests because of dissimilar delamination depths from these two faces (depths of 1, 2, 3, and 4 cm from front-face and depths of 5, 6, 7, and 8 cm from the back-face). In particular, the specimen was heated uniformly in various heating regimes, i.e., 3, 5, 10, 15, and 20 min from the back-face and 5, 10, 15, 20, 25, 30, and 40 min from the front-face. Therefore, 36 cases in total were considered in this study. To conduct the test in each case, the concrete specimen was heated (heating duration), then it was cooled (cooling time after the halogen lamps were turned off) under the environmental conditions in the laboratory. The surface temperature was recorded during not only the heating time but also the cooling duration at a capturing frequency of 0.5 Hz using a long-wavelength IR camera (FLIR SC660) with a thermal sensitivity of 0.03 °C [56]. The technical specifications of the IR camera are described in detail in our previous studies [14,35,36].

The surface temperature of structure can be affected by several factors such as reflected temperature, ambient conditions, non-uniform heating, subsurface defect, especially emissivity variation. The emissivity shows the ability that the object surface can emit the energy by radiation related to a black body that is an ideal surface with the emissivity of 1.0. The emissivity is a unitless quantity in which it is always less than unity for a real surface. In fact, the emissivity is not a constant that depends on the surface condition, measurement angle, temperature as well as wavelengths. However, the emissivity was usually fixed as a constant during the experiment in previous studies. In this work, for simplify, the emissivity of concrete and CFRP are considered as 0.95 and 0.97, respectively, during all tests as mentioned in previous studies [13,57,58,59,60].

## 4. Discussion of Results

### 4.1. Detection of Delamination

In this section, we examine the capability of detecting delaminations at the core of a concrete structure strengthened by a single sheet of CFRP. A delamination is determined to be detectable or undetectable based on both the qualitative assessment from the observation of thermal images and quantitative assessment from the absolute contrast value in conjunction with signal-to-noise ratio criterion. In terms of the quantitative assessment, a delamination is detected if the absolute contrast (Δ*T*) is equal to or higher than the noise equivalent temperature difference (NETD = 0.03 °C) of the IR camera and the SNR is higher than zero, whereas it is not detected if Δ*T* is less than the NETD and/or a negative SNR is obtained [29,61,62].

**Figure 4:**
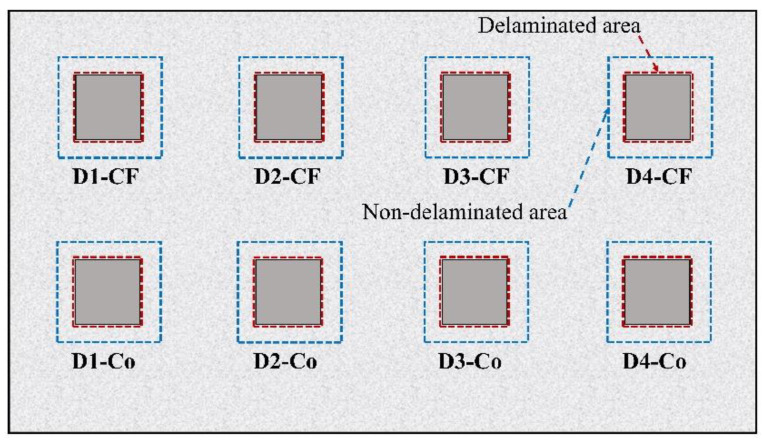
Selection of delaminated and non-delaminated areas.

The absolute contrast and the SNR of delaminations are computed using Equation (4) and Equations (5), respectively. As presented in Figure 4, the area of region of interest (ROI) covering the defect (so-called delaminated area) is used to calculate *T_de_*(*t*) and *S* while *T_nde_*(*t*), *N*, and $\sigma_{noise}$ are taken from of a ROI as a perimeter around the imitating deterioration (so-called non-delaminated area). In the present study, eight delaminations have the same dimensions of 10 cm × 10 cm × 1 cm, both signal and noise areas are thus selected with the same sizes for all defects. Delaminated and non-delaminated areas are expressed corresponding to red and blue squares as shown in Figure 4.

**Figure 5:**
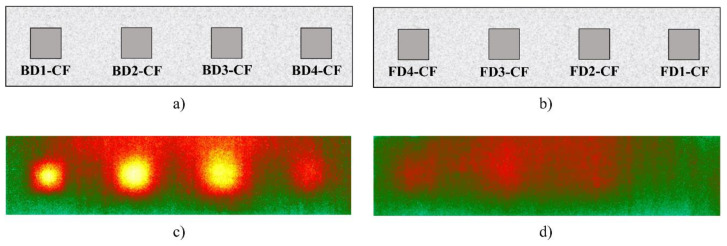
(**a**) Defects locations from back-face; (**b**) defects locations from front-face; (**c**) thermal image after 10 min of cooling under 15-min heating from back-face; (**d**) thermal image after 40 min of cooling under 25 min heating from front-face.

Figure 5a,b illustrates the location of the delamination from the back- and front-faces, respectively. Moreover, Figure 5c,d shows the thermal images taken during cooling corresponding to the case of the 15-min heating from the back-face and 25-min heating from the front-face. It can be observed from these images that, except for delamination FD1-CF (depth = 8 cm), defects with depths equal to or smaller than 7 cm were discovered under the observation of human eyes (qualitative assessment). It should be noted that the depths are 1, 2, 3, and 4 cm from the back-face while delaminations are located more deeply at 5, 6, 7, and 8 cm from the front-face (see Figure 2 and Table 1). As a result, in terms of qualitative assessment, it is seen that delaminations are observed more clearly in Figure 5c than those in Figure 5d even a longer heating time is provided for the back-face compared to the front-face due to the effect of defect depths. Therefore, a statement can be given since deeper delaminations can be observed less clearly than those at shallower depths.

**Figure 6:**
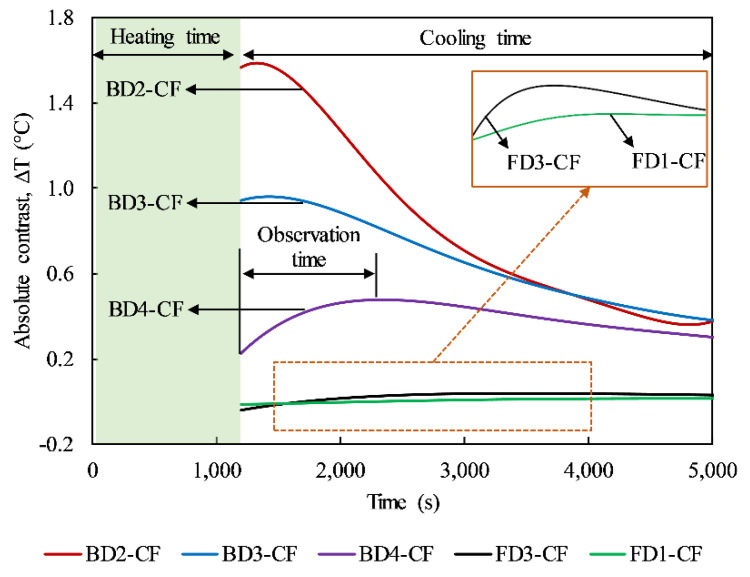
Absolute contrast curves for delaminations with 20 min heating.

In this work, a thermal image was captured every 2 s. Therefore, the absolute contrast can be analyzed in a time-sequence for each delamination, as shown in Figure 6. This can be used not only for evaluating the delamination detection based on the absolute contrast but also for determining the observation time (*OBT*). In Figure 6, a fifth-order polynomial function is used to fit the Δ*T* curves that helps to reduce the noise. It can be seen that after turning off the lamps, Δ*T* increases and reaches a peak before decreasing. The *OBT* is defined as the duration from the start of the cooling time to the peak of the Δ*T* curve. The *OBT* is an important factor in the prediction of the depth of defects and is discussed in detail in Section 4.3.

**Figure 7:**
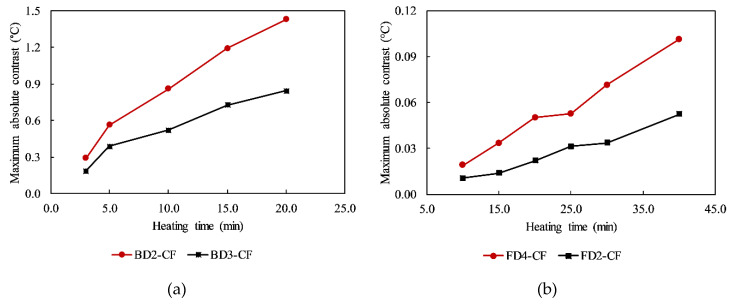
Maximum absolute contrast values of delaminations: (**a**) BD2-CF and BD3-CF; (**b**) FD4-CF and FD2-CF.

The maximum Δ*T* values of some delaminations in the CSC areas of the specimen are shown in Figure 7. It can be seen that for the same heating time, a shallower defect can produce a higher contrast on the image than a deeper one. For example, under 10-min heating, the maximum Δ*T*s of delaminations BD2-CF and BD3-CF are 0.86 and 0.52 °C, respectively. In addition, the longer is the heating time, the higher is the maximum Δ*T* that can be obtained. For instance, the maximum Δ*T*s are 0.02, 0.05, and 0.07 °C corresponding to 10 min, 20 min, and 30 min heating regimes for delamination FD4-CF, respectively. Therefore, in terms of absolute contrast values, it can be concluded that a delamination more deeply located is detected less clearly than a delamination closer to the structure’s surface, and a longer heating regime can better indicate the occurrence of a delamination compared to a shorter heating time.

In particular, almost every delamination could be detected from the back-face under 3 min and 5 min heating except delamination BD4-CF, at a depth of 4 cm, with maximum Δ*T*s less than 0.03 °C. With longer heating times (10, 15, and 20 min), all defects from the back-face (depth ≤ 4 cm) could be detected clearly (maximum Δ*Ts* > 0.03 °C and SNR > 0 dB). In addition, delaminations FD4-CF (Z = 5 cm), FD3-CF (Z = 6 cm), and FD2-CF (Z = 7 cm) become detectable when the structure is heated for 15, 20, and 25 min, respectively (maximum Δ*T*s > 0.03 °C and SNR > 0 dB). However, the deepest delamination (FD1-CF) might not be identifiable on the thermal image even if the specimen is heated for 40 min and a maximum Δ*T* of 0.033 °C but a negative SNR of −0.653 dB is obtained.

### 4.2. Effect of CFRP on Delamination Detection

**Figure 8:**
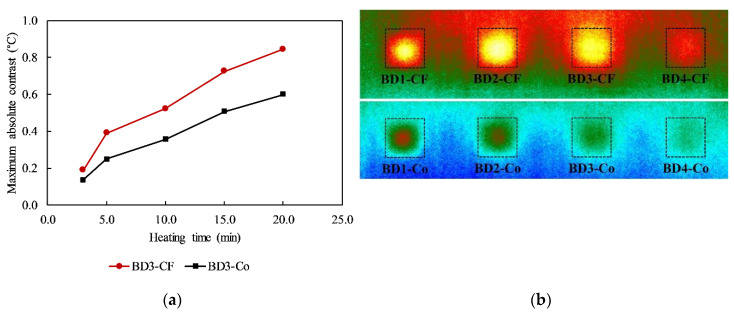
Effect of CFRP: (**a**) maximum absolute contrasts of delaminations BD3-CF and BD3-Co; (**b**) thermal image after cooling for 20 min in case of 15 min heating.

Figure 8a shows the maximum Δ*T*s of delaminations (BD3-CF in the CSC area and BD3-Co in the CWC area) with the same parameters (listed in Table 1) under different heating times. Moreover, the SNR is also provided following the absolute contrast value.

With the identical experimental condition, a higher maximum Δ*T* was obtained regarding the delaminations in the CSC structure compared to those in the CWC structure. For example, under 15-min heating, the maximum Δ*T*s were 0.51 (SNR = 6.02 dB) and 0.73 °C (SNR = 8.66 dB) corresponding to delaminations BD3-Co and BD3-CF, respectively. This phenomenon also can be observed clearly for other defects as shown in Figure 8b. This implies that delaminations in the CSC structure appear more distinctly on thermal images than those in the CWC structure. This might be explained in that the CFRP surface can absorb more energy from lamps than the concrete surface (the emissivity of CFRP is higher than concrete).

The difference in the maximum Δ*T* between delaminations in the CSC and CWC structures show an increasing trend with an increase in heating time. For instance, the maximum Δ*T* differences are 0.16 (1.88 dB in SNR criterion) and 0.24 °C (2.41 dB in SNR criterion) corresponding to 10 and 20 min heating, respectively.

### 4.3. Prediction of Delamination Depth

The depth of a delamination (*Z*) can be computed as the product of the nondimensional prefactor (*k*), root mean square of the thermal diffusivity (*α*), and root mean square of the observation time (*OBT*) as shown in Equation (13) [18].

(13) $$Z=k\sqrt{\alpha \times OBT}$$

From a practical viewpoint, the relationship between the squared depth of the delamination and observation time is usually utilized as shown in Equation (14), where *α_e_* is considered as the empirical thermal diffusivity.

(14) $$Z^{2}=\alpha_{e}\times OBT$$

**Figure 9:**
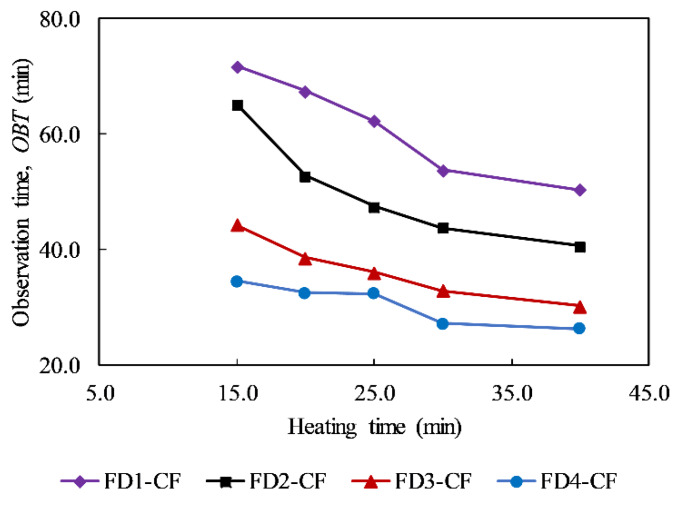
Observation time of delaminations.

It can be seen that the *OBT* is a valuable factor in predicting the delamination depth. The *OBT* is estimated from Δ*T* curves as shown in Figure 6. Figure 9 depicts the *OBTs* of delaminations at depths of 5, 6, 7, and 8 cm. It is stated that a longer heating time leads to a reduction in the *OBT*. For example, the *OBTs* of delamination FD4-CF are 34.6 min and 27.3 min corresponding to 15 and 30 min heating regimes, respectively. In addition, deeper delaminations take a longer time to reach a peak Δ*T* value. For instance, in the case of 30 min heating, the *OBT*s of delaminations FD4-CF, FD3-CF, FD2-CF, and FD1-CF are 27.4, 33.0, 43.9, and 53.8 min, respectively.

**Figure 10:**
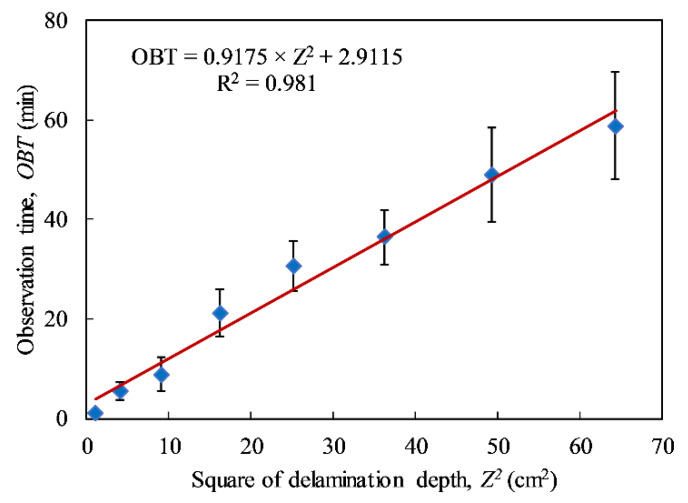
Relationship between the observation time under all cases of heating times and square of delamination depth.

The relationship between the squares of known defect depths and mean values of *OBT*s in all cases of heating times is plotted in Figure 10. A higher standard deviation can be achieved in the case of a deeper delamination. The standard deviations range from 0.6 to 10.7 min when depths increase from 1 to 8 cm. A regression line is used to fit the data, and the reliability of the data set is proved with a high coefficient of determination (*R*^2^ = 0.981). Thereafter, the intercept is set to zero and the new equation is determined as *OBT* = 0.9849 × *Z*^2^. Hence, the empirical thermal diffusivity coefficient can be estimated to be 1.0153 cm^2^/min (*α_e_* = 1/0.9849). It is noted that the thermal diffusivity is about 3.7 and 0.53 cm^2^/min corresponding to CFRP and concrete as mentioned in Maldague’s book [18]. The thermal diffusivity proposed for concrete strengthened with one CFRP layer is higher than concrete and lesser than CFRP.

**Figure 11:**
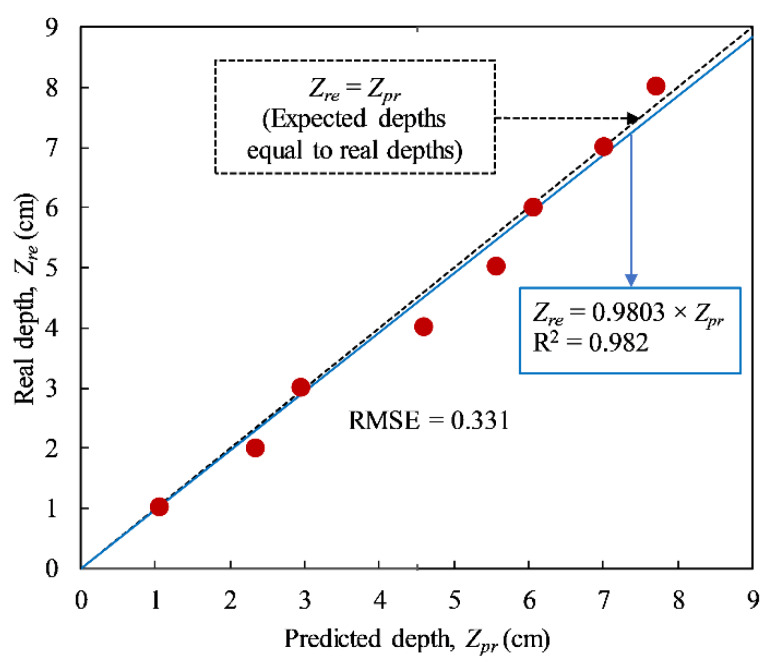
Prediction of delamination depths in CSC structure under all cases of heating times.

The relationship in Equation (14) and the proposed *α_e_* factor are used to predict the depth of delaminations. The mean values of the predicted depths under all cases of heating times in this study are shown in Figure 11. The blue solid line indicates the idealized line, while the black dashed line (empirical line) is the linear regression line between the expected and real depths. The difference in the slopes of the idealized and empirical lines is only approximately 2%. This proves that the obtained thermal diffusivity coefficient can be used to predict delamination depths in the CSC girder having similarities compared to the present study during field inspections. Because the data set of *OBT*s used to forecast delamination depths in Figure 11 are the same as those employed to estimate the empirical coefficient, the formula of the linear regression line therefore should be tested and confirmed further with other case studies.

**Figure 12:**
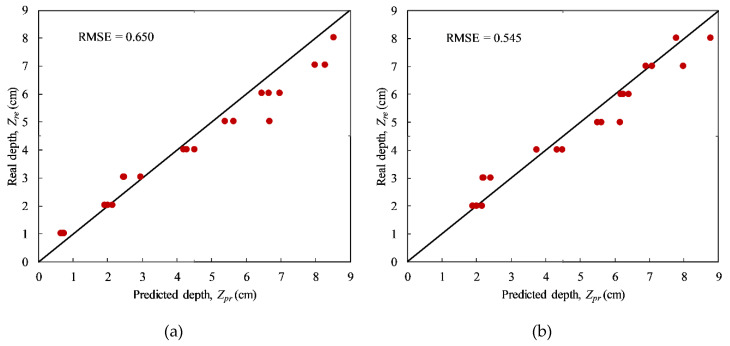
Predicted defect depths under different heating times: (**a**) 15 min and (**b**) 20 min.

Figure 12 shows the predicted depths of delaminations in the CSC structure under two different heating times, i.e., 15 and 20 min since the accuracy of the entire predicted depths (depths from 1 to 8 cm) can be estimated. The root mean square error (RMSE) is used as an indicator to evaluate the accuracy of the forecasted depths. Particularly, the RMSEs are 0.650 and 0.545 corresponding to the 15 min and 20 min heating cases, respectively. Because delaminations tested from the front-face could not be detected with the heating time equal to or lesser than 10 min, under 3-min, 5-min, and 10-min heating the accuracy of forecasted depths can be only considered for delaminations from the back-face (depths from 1 to 4 cm). It is found that the RMSEs are 0.69, 0.68, and 0.42 corresponding 3-min, 5-min, and 10-min heating. In addition, under the heating times of 25, 30, and 40 min, only the depth of delaminations from the front-face (depth from 5 to 8 cm) can be evaluated with the experimental data set in the present study. Namely, the RMSEs are 0.63, 0.64, and 0.50 corresponding 25, 30, and 40 min heating. Therefore, it can be stated that the longer is the heating time, the higher is the achieved accuracy.

### 4.4. Enhancement of Delamination Detection Using Post-Processing Algorithms

In the detection of defects based on thermography, such as with the SHT method, the accurate selection of the delaminated and non-delaminated areas is critical so that the absolute contrast and observation time can be determined correctly. In this study, the delaminated and non-delaminated areas are pre-known; therefore, the region selection is not a difficult task. However, this problem poses a challenge in the investigation of actual structures and potential defects need to be identified as accurately as possible. Therefore, the application of individual image processing methods or their combination should be conducted to avoid confusion or misdetection of defects.

In the present study, three post-processing algorithms are considered including absolute contrast (ABC), pulsed phase thermography (PPT), and thermal signal reconstruction (TSR). ABC is a simple method applied to analyze the data in previous sections. In this research, the results obtained on applying the ABC method are considered as the standard indicators used to assess the efficiency of the TSR and PPT methods. The theory of these algorithms is presented in detail in Section 2.

**Figure 13:**
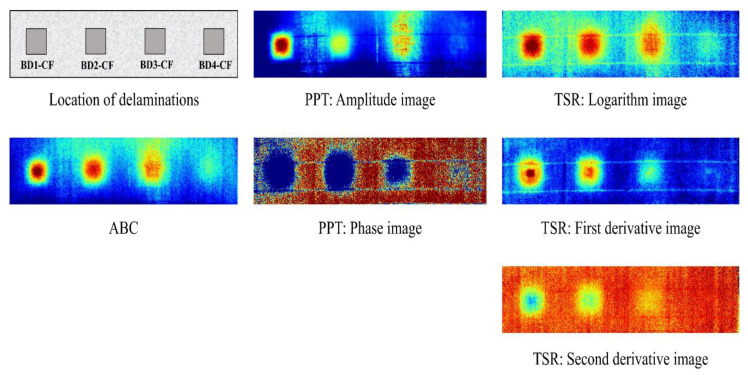
Thermal images using different algorithms (data used in the case of 10-min heating from back-face).

Figure 13 shows the thermal images obtained using the ABC (at 12 min in cooling time), PPT (at the frequency of 0.38 × 10^−3^ Hz), and TSR methods (logarithm, first derivative and second derivative images at 13, 8, and 12 min in the cooling time, respectively) for the data in the case of 10-min heating. It should be noted that the logarithm depicted in Equation (8) and the first and second derivative data from Equation (9) are utilized in the TSR method, whereas amplitude and phase data are used in the PPT method. It is seen that the noise around the defects can be reduced significantly using the PPT and TSR methods in some cases when compared to the ABC approach, especially for delaminations BD2-CF and BD3-CF in the phase, first, and second derivative images. Therefore, several defects might be observed with more certainty.

The maximum SNRs obtained from different algorithms under 5, 10, and 15 min heating are presented in Table 2. It is seen from Table 2 that the TSR and PPT algorithms can be applied to increase the SNR (the goodness of contrast) of delamination in some cases as compared to the ABC approach. For example, the maximum SNRs of delamination BD2-CF are 9.74 and 9.49 dB on applying the PPT (phase data) and TSR methods (first derivative data), respectively, while it is only 4.98 dB on applying the ABC method in the case of heating for 5 min. However, individual algorithms (TSR or PPT) cannot increase the maximum SNR of all delaminations, which might not be expected. For instance, on using TSR, the maximum SNR of delamination BD1-CF is not enhanced, yet better results are achieved for defect BD2-CF (an increase in the SNR from 4.98 to 9.49 dB using first derivative data) and for delamination BD3-CF (SNR enhanced from 1.90 to 4.61 dB using logarithmic data) in comparison with the ABC method under 5-min heating. Furthermore, although PPT is applied, the SNR of defect BD3-CF is decreased (3.46 and 1.82 dB corresponding to phase and amplitude data) compared to the ABC method (5.08 dB) if 10-min heating is provided.

The maximum SNR and enhancement level (in percentage) obtained using a combination of the PPT and TSR methods are listed in two last columns of Table 2. The combination means that the data is firstly analyzed by individual methods, i.e., PPT and TSR, then, the best result of the processing process are taken as the maximum value from an outcome combination of both two methods. It can be observed that these two image processing methods can increase the SNR of all delaminations compared to the ABC method in the case of 5 min heating, while half of the delaminations (BD1-CF and BD4-CF) produce poorer outcomes in comparison with the ABC method under 10 and 15 min heating. It is found that the effective detection of delaminations can be obtained even through simple methods such as ABC. Therefore, it is recommended that several image processing methods be applied to analyze the data, and the combination of their results be used to more accurately identify delaminations on the thermal image. On using this combination, the confusion and misdetection phenomenon in the core of the CSC structure might be reduced or avoided.

In addition, by comparison of the results from PPT (maximum SNR of amplitude and phase) and TSR (maximum SNR of logarithm, first derivative and second derivative) as shown in Table 2, under 5-min heating, almost delaminations obtain the better contrast by using PPT than those using TSR. However, when the heating time is expanded, i.e., 10 and 15 min, the PPT achieves a poor quality than TSR. This can be explained that the PPT technique is an effective method to enhance the visibity of defects in the IRT technique especially when the heating time is small.

## 5. Conclusions

In the present study, an effort was made to detect delaminations at the core of a specimen assumed to be the surface of a concrete girder strengthened with one CFRP layer not exposed directly to the sunlight using a thermography-based method, i.e., step heating thermography. From results provided in this study, the following conclusions are given:

1. If a similar experiment setup is applied and heating times up to 40 min are provided, delamination with a depth equal to or less than 7.0 cm in CSC girders can be accurately identified using the step heating thermography method.

2. The absolute contrast above a delamination indicating its detectability increases significantly under the effect of the CFRP material installed on the structure’s surface. Hence, if the tested area covers both CSC and CWC regions, with the identical parameters, delamination in a CSC region can be observed more definitively in comparison with those in areas without the strengthening.

3. On using the experimental approach, the empirical thermal diffusivity coefficient, which has not been provided previously, is proposed (*α_e_* = 1.0153 cm^2^/min) in this study, as the depths of delamination can be predicted with a high accuracy. Thus, it is recommended that this provided factor be applied to the field evaluations of CSC girders having similarities with this study.

4. The effectiveness of post-processing algorithms including pulsed phase thermography and thermal signal reconstruction are examined. The detection of delamination in the core of CSC girders can be enhanced considerably in some cases by using a combination of results from two abovementioned algorithms compared to the absolute contrast method. Moreover, when the heating time provided is increased, the TSR becomes more effectively than the PPT algorithm.

## Figures and Tables

**Table 1:** Characteristics of delaminations.

Defect	Depth (cm)	Testing Face	Structure	Defect	Depth (cm)	Testing Face	Structure
BD1-CF	1.0	Back	CWC	FD1-CF	8.0	Front	CWC
BD1-Co	1.0	Back	CSC	FD1-Co	8.0	Front	CSC
BD2-CF	2.0	Back	CWC	FD2-CF	7.0	Front	CWC
BD2-Co	2.0	Back	CSC	FD2-Co	7.0	Front	CSC
BD3-CF	3.0	Back	CWC	FD3-CF	6.0	Front	CWC
BD3-Co	3.0	Back	CSC	FD3-Co	6.0	Front	CSC
BD4-CF	4.0	Back	CWC	FD4-CF	5.0	Front	CWC
BD4-Co	4.0	Back	CSC	FD4-Co	5.0	Front	CSC

**Table 2:** Maximum SNR (dB) of defects using different algorithms under 5, 10, and 15 min heating from back-face.

Defects	ABC Method	PPT Method		TSR Method			Combination of PPT and TSR Methods	
		Amplitude	Phase	Logarithm	First Derivative	Second Derivative	Maximum SNR	Increment Compared to ABC (%)
5 min heating								
BD1-CF	15.36	16.31	9.79	13.52	8.87	4.00	16.31	6.22
BD2-CF	4.98	2.01	9.74	6.83	9.49	6.11	9.74	95.42
BD3-CF	1.90	1.61	1.17	4.61	0.19	−1.87	4.61	142.60
BD4-CF	−6.01	−10.75	−4.07	−11.07	−6.00	−10.80	−4.07	32.23
10 min heating								
BD1-CF	18.19	16.56	10.23	16.56	15.15	12.62	16.56	−8.95
BD2-CF	10.54	5.14	13.91	10.87	14.06	10.68	14.06	33.39
BD3-CF	5.08	1.82	3.46	5.89	4.58	2.85	5.89	16.11
BD4-CF	2.36	0.80	0.96	0.21	1.58	−0.19	1.58	−33.18
15 min heating								
BD1-CF	20.37	18.77	10.96	18.78	13.55	15.06	18.78	−7.82
BD2-CF	12.10	6.79	13.32	11.43	16.02	13.11	16.02	32.39
BD3-CF	8.66	5.39	0.67	9.26	5.82	5.71	9.26	7.01
BD4-CF	4.80	1.76	3.68	2.89	4.37	1.72	4.37	−9.01

## References

[1] Hiasa S., Birgul R., Catbas F.N. (2017). Investigation of effective utilization of infrared thermography (IRT) through advanced finite element modeling. Constr. Build. Mater..

[2] Tran Q.H. (2019). Quantitative Evaluation of Deteriorations for Concrete Bridge Structures using Square Pulse Thermography. Ph.D. Thesis.

[3] Hiasa S. (2016). Investigation of Infrared Thermography for Subsurface Damage Detection of Concrete Structures. Ph.D. Thesis.

[4] Chen F., Baji H., Li C.-Q. (2018). A comparative study on factors affecting time to cover cracking as a service life indicator. Constr. Build. Mater..

[5] Su R.K.L., Zhang Y. (2015). A double-cylinder model incorporating confinement effects for the analysis of corrosion-caused cover cracking in reinforced concrete structures. Corros. Sci..

[6] Zhang Y., Su R.K.L. (2019). Concrete cover delamination model for non-uniform corrosion of reinforcements. Constr. Build. Mater..

[7] Xi X., Yang S. (2017). Time to surface cracking and crack width of reinforced concrete structures under corrosion of multiple rebars. Constr. Build. Mater..

[8] Klaiber F.W., Dunker K.F., Wipf T.J., Sanders W.W. (1988). Methods of Strengthening Existing Highway Bridges, Report 293.

[9] Chajes M., Rollins T., Dai H., Murphy T. (2019). Report on Techniques for Bridge Strengthening, FHWA-HIF-18-041.

[10] ElSafty A., Graeff M. (2012). The Repair of Damaged Bridge Girders with Carbon-Fiber-Reinforced Polymer “CFRP” Laminates, BDK82 977-03.

[11] (2017). ACI Committee 440 440.2R-17: Guide for the Design and Construction of Externally Bonded FRP Systems for Strengthening Concrete Structures.

[12] Tashan J., Al-Mahaidi R. (2014). Bond defect detection using PTT IRT in concrete structures strengthened with different CFRP systems. Compos. Struct..

[13] Vaghefi K. (2013). Infrared Thermography Enhancements for Concrete Bridge Evaluation. Ph.D. Thesis.

[14] Huh J., Mac V.H., Tran Q.H., Lee K.-Y., Lee J.-I., Kang C. (2018). Detectability of Delamination in Concrete Structure Using Active Infrared Thermography in Terms of Signal-to-Noise Ratio. Appl. Sci..

[15] Forde M. (2010). International practice using NDE for the inspection of concrete and masonry arch bridges. Bridg. Struct..

[16] (2013). ASTM D4788-03, Standard Test Method for Detecting Delaminations in Bridge Decks Using Infrared Thermography.

[17] Zhang H., Yang R., He Y., Foudazi A., Cheng L., Tian G. (2017). A Review of Microwave Thermography Nondestructive Testing and Evaluation. Sensors.

[18] Maldague X. (2001). Theory and Practice of Infrared Thermography for Nondestructive Testing.

[19] Milovanovic B., Pečur I.B. (2016). Review of Active IR Thermography for Detection and Characterization of Defects in Reinforced Concrete. J. Imaging.

[20] Janků M., Cikrle P., Grošek J., Anton O., Stryk J. (2019). Comparison of infrared thermography, ground-penetrating radar and ultrasonic pulse echo for detecting delaminations in concrete bridges. Constr. Build. Mater..

[21] Tashan J., Al-Mahaidi R. (2014). Detection of cracks in concrete strengthened with CFRP systems using infra-red thermography. Compos. Part B Eng..

[22] Gu J.C., Unjoh S., Naito H. (2020). Detectability of delamination regions using infrared thermography in concrete members strengthened by CFRP jacketing. Compos. Struct..

[23] Lai W.W., Kou S., Poon C.S., Tsang W., Lai C. (2010). Characterization of the deterioration of externally bonded CFRP-concrete composites using quantitative infrared thermography. Cem. Concr. Compos..

[24] Liu B., Zhang H., Fernandes H.C., Maldague X. (2016). Quantitative Evaluation of Pulsed Thermography, Lock-in Thermography and Vibrothermography on Foreign Object Defect (FOD) in CFRP. Sensors.

[25] Shi Q., Liu J., Liu W., Wang F., Wang Y. (2019). Barker-coded modulation laser thermography for CFRP laminates delamination detection. Infrared Phys. Technol..

[26] Shardakov I., Shestakov A.P., Bykov A. (2016). Delamination of carbon-fiber strengthening layer from concrete beam during deformation (infrared thermography). Frattura ed Integrità Strutturale.

[27] Wu S., Gao B., Yang Y., Zhu Y., Burrascano P., Laureti S., Ricci M., Wang Y. (2019). Halogen optical referred pulse-compression thermography for defect detection of CFRP. Infrared Phys. Technol..

[28] Lai W.W., Lee K., Kou S., Poon C., Tsang W. (2012). A study of full-field debond behaviour and durability of CFRP-concrete composite beams by pulsed infrared thermography (IRT). NDT E Int..

[29] Larsen C.A. (2011). Document Flash Thermography. Master’s Thesis.

[30] D’Accardi E., Palumbo D., Tamborrino R., Galietti U. (2018). A Quantitative Comparison Among Different Algorithms for Defects Detection on Aluminum with the Pulsed Thermography Technique. Metals.

[31] Almond D.P., Pickering S.G. (2012). An analytical study of the pulsed thermography defect detection limit. J. Appl. Phys..

[32] Zheng K., Chang Y.-S., Wang K.-H., Yao Y. (2015). Improved non-destructive testing of carbon fiber reinforced polymer (CFRP) composites using pulsed thermograph. Polym. Test..

[33] Lizaranzu M., Lario A., Chiminelli A., Amenabar I. (2015). Non-destructive testing of composite materials by means of active thermography-based tools. Infrared Phys. Technol..

[34] Tashan J., Al-Mahaidi R., Mamkak A. (2015). Defect size measurement and far distance infrared detection in CFRP-concrete and CFRP-steel systems. Aust. J. Struct. Eng..

[35] Mac V.H., Tran Q.H., Huh J., Doan N.S., Kang C., Han D. (2019). Detection of Delamination with Various Width-to-depth Ratios in Concrete Bridge Deck Using Passive IRT: Limits and Applicability. Materials.

[36] Tran Q.H., Huh J., Mac V.H., Kang C., Han D. (2018). Effects of rebars on the detectability of subsurface defects in concrete bridges using square pulse thermography. NDT E Int..

[37] Vavilov V. (2007). Pulsed thermal NDT of materials: Back to the basics. Nondestruct. Test. Eval..

[38] Bauer E., Pavón E., Pereira C.H.F., Nascimento M.L.M. (2016). Criteria for Identification of Ceramic Detachments in Building Facades with Infrared Thermography. Recent Developments in Building Diagnosis Techniques. Building Pathology and Rehabilitation.

[39] Brooke C. (2018). Thermal Imaging for the Archaeological Investigation of Historic Buildings. Remote. Sens..

[40] Cheng C.-C., Cheng T.-M., Chiang C.-H. (2008). Defect detection of concrete structures using both infrared thermography and elastic waves. Autom. Constr..

[41] Pilla M., Klein M., Maldague X., Salerno A. New Absolute Contrast for pulsed thermography. Proceedings of the 2002 International Conference on Quantitative InfraRed Thermography; QIRT Council.

[42] González D., Ibarra-Castanedo C., Pilla M., Klein M., Lopez-Higuera J., Maldague X. Automatic interpolated differentiated absolute contrast algorithm for the analysis of pulsed thermographic sequences. Proceedings of the 2004 International Conference on Quantitative InfraRed Thermography; QIRT Council.

[43] Venter G. (2016). Non-destructive testing with transient thermography on composite materials. R D J. S. Afr. Inst. Mech. Eng..

[44] Vavilov V.P., Burleigh D.D. (2015). Review of pulsed thermal NDT: Physical principles, theory and data processing. NDT E Int..

[45] Ibarra-Castanedo C., Benítez H.D., Maldague X.P.V., Bendada A. Review of thermal-contrast-based signal processing techniques for the nondestructive testing and evaluation of materials by infrared thermography. Proceedings of the International Workshop on Imaging NDE.

[46] Tran Q.H., Huh J., Kang C., Lee B.Y., Kim I.-T., Ahn J.-H. (2018). Detectability of Subsurface Defects with Different Width-to-Depth Ratios in Concrete Structures Using Pulsed Thermography. J. Nondestruct. Eval..

[47] Tran Q.H., Han D., Kang C., Haldar A., Huh J. (2017). Effects of Ambient Temperature and Relative Humidity on Subsurface Defect Detection in Concrete Structures by Active Thermal Imaging. Sensors.

[48] Huh J., Tran Q.H., Lee J.-H., Han D., Ahn J.-H., Yim S. (2016). Experimental Study on Detection of Deterioration in Concrete Using Infrared Thermography Technique. Adv. Mater. Sci. Eng..

[49] Hidalgo-Gato R., Andrés J.R., López-Higuera J.M., Madruga F.J. (2013). Quantification by Signal to Noise Ratio of Active Infrared Thermography Data Processing Techniques. Opt. Photon- J..

[50] Zheng K., Chang Y.-S., Yao Y. (2015). Defect detection in CFRP structures using pulsed thermographic data enhanced by penalized least squares methods. Compos. Part B: Eng..

[51] Mabry N.J., Peters K., Seracino R. (2015). Depth Detection of Bond Defects in Multilayered Externally Bonded CFRP-to-Concrete Using Pulse Phase Thermography. J. Compos. Constr..

[52] Lu X., Liao G., Zha Z., Xia L., Shi T. (2011). A novel approach for flip chip solder joint inspection based on pulsed phase thermography. NDT E Int..

[53] Pollock D.G., Dupuis K.J., Lacour B., Olsen K.R. (2008). Detection of Voids in Prestressed Concrete Bridges Using Thermal Imaging and Ground-Penetrating Radar.

[54] Arndt R.W. (2010). Square pulse thermography in frequency domain as adaptation of pulsed phase thermography for qualitative and quantitative applications in cultural heritage and civil engineering. Infrared Phys. Technol..

[55] Cotič P., Kolarič D., Bosiljkov V.B., Bosiljkov V., Jagličić Z. (2015). Determination of the applicability and limits of void and delamination detection in concrete structures using infrared thermography. NDT E Int..

[56] FLIR System Inc. (2014). SC660 Catalog, Technical Data of FLIR SC660 Infrared Camera.

[57] (2013). ACI Committee 228 228.2R-13: Report on Nondestructive Test Methods for Evaluation of Concrete in Structures.

[58] Starnes M. (2002). Development of Technical Bases for Using Infrared Thermography for Nondestructive Evaluation of Fiber Reinforced Polymer Composites Bonded to Concrete. Ph.D. Thesis.

[59] Grammatikos S., Kordatos E., Matikas T., David C., Paipetis A. (2014). Current injection phase thermography for low-velocity impact damage identification in composite laminates. Mater. Des..

[60] Omar T., Nehdi M.L., Zayed T. (2018). Infrared thermography model for automated detection of delamination in RC bridge decks. Constr. Build. Mater..

[61] Vavilov V.P., Chulkov A., Derusova D.A., Pan Y. (2016). Thermal NDT research at Tomsk Polytechnic University. Quant. Infrared Thermogr. J..

[62] Svantner M., Muzika L., Chmelík T., Skala J. (2018). Quantitative evaluation of active thermography using contrast-to-noise ratio. Appl. Opt..

